# Quality of Post Arrest Care Does Not Differ by Time of Day at a Specialized Resuscitation Center

**DOI:** 10.1097/MD.0000000000000664

**Published:** 2015-04-10

**Authors:** Thomas Uray, Fritz Sterz, Christoph Weiser, Wolfgang Schreiber, Alexander Spiel, Andreas Schober, Peter Stratil, Florian B. Mayr

**Affiliations:** From the Department of Emergency Medicine (TU, FS, CW, WS, Alexander Spiel, Andreas Schober, PS), Medical University of Vienna, Vienna, Austria; and CRISMA Center (FBM), Department of Critical Care Medicine, University of Pittsburgh, Pittsburgh, Pennsylvania, USA.

## Abstract

Previous studies suggest worse outcomes after out-of-hospital cardiac arrest (OHCA) at night. We analyzed whether patients admitted after nontraumatic OHCA to a resuscitation center received the same quality post arrest care at day and night and whether quality of care affected clinical outcomes.

We analyzed data of OHCA patients with return of spontaneous circulation admitted to the Vienna general hospital emergency department between January 2006 and May 2013. Data reported include admission time (day defined from 8 am to 4 pm based on staffing), time to initiation of hypothermia, and door-to-balloon time in patients with ST-elevation myocardial infarction. Survival and cognitive performance at 12 months were assessed.

In this retrospective observational study, 1059 patients (74% males, n = 784) with a mean age of 58 ± 16 years were analyzed. The vast majority was treated with induced hypothermia (77% of day vs. 79% of night admissions, *P* = 0.32) within 1 hour of admission (median time admission to cooling 27 (confidence interval [CI]: 10–60) vs. 23 (CI: 11–59) minutes day vs. night, *P* = 0.99). In 298 patients with ST-elevation myocardial infarction, median door-to-balloon time did not differ between day and night admissions (82 minutes, CI: 60 to 142 for day vs. 86 minutes, CI: 50 to 135 for night, *P* = 0.36).

At 12 months, survival was recorded in 238 of 490 day and 275 of 569 night admissions (49% vs. 48%, *P* = 0.94%), and a good neurologic outcome was recorded in 210 of 490 day and 231 of 569 night admissions (43% vs. 41%, *P* = 0.46).

Patients admitted to our department after OHCA were equally likely to receive timely high-quality postresuscitation care irrespective of time of day. Survival and good neurologic outcome at 12 months did not differ between day and night admissions. Our results may support the concept of specialized post arrest care centers.

## INTRODUCTION

Cardiac arrest is a major health problem worldwide accounting for more than 675 000 cases per year in Europe and the United States together.^1–3^ The overall survival rate remains poor at approximately 10%.^4^ The odds of survival are increased by immediate activation of the emergency medical services, early cardiopulmonary resuscitation (CPR), rapid defibrillation, effective advanced life support, and integrated postcardiac arrest care.^5–9^ Several recent retrospective studies reported significantly lower survival rates for out-of-hospital cardiac arrest (OHCA) patients at night vs. day.^10–13^ These studies adjusted for some prehospital components of care such as early defibrillation, bystander CPR and time to emergency medical services arrival, as well as patient characteristics including age and initial rhythm, but had no information on the quality of in-hospital post arrest care. Previous studies have identified hospitals with a higher volume of cardiac arrest patients, percutaneous coronary intervention (PCI) capability, and teaching status to have better OHCA outcomes.^1,14–17^ In particular, early PCI and induced hypothermia (IH) are associated with improved survival and functional recovery after OHCA.^2,17,18^ However, these interventions may not be as readily available during nighttime because of reduced staffing.

We, therefore, aimed to understand whether previously reported worse outcomes after OHCA arrest at nighttime are, in part, due to differences in post arrest care. We analyzed the quality of in-hospital post arrest care by time of day in a cohort of OHCA survivors admitted to our institution, a center specialized in post arrest care and further explored effects on survival and neurologic outcome at 12 months.

## METHODS

### Study Design and Setting

We conducted a retrospective observational analysis of a cardiac arrest data registry, which was approved by the institutional ethical review board. This trial qualified for exception from informed consent requirements for emergency research, as outlined in the applicable national laws of Austria. This trial complies with the Declaration of Helsinki.

The registry includes information on all cardiac arrest patients admitted to the Department of Emergency Medicine, Vienna General Hospital, a 2200-bed quaternary care facility with integrated intensive care unit (ie, resuscitation center) affiliated with the Medical University of Vienna, Vienna, Austria. This emergency department handles around 88 000 adult nontraumatic patients every year, including 1300 critically ill patients and 250 cardiac arrest patients. At our institution PCI is available 24/7, with a 30-minute call-in-time at night, and a hospital-based team around the clock on weekends.

The city of Vienna with its 1.8 million inhabitants is covered by a physician-based emergency medical service with 18 ambulances and 1 emergency helicopter. Of these, 7 ambulances and the helicopter are staffed with emergency physicians who treat approximately 700 OHCA annually.^19^

All patients 18 years or older who suffered a nontraumatic OHCA and regained restoration of spontaneous circulation (ROSC) between January 1, 2006 and May 31, 2013 were included in our analysis. Data were collected according to the Utstein guidelines.^20^ Patient data included sex, age, location of cardiac arrest (home or public place), witnessed or nonwitnessed cardiac arrest, performance of basic life support (BLS) by bystanders, first documented electrocardiogram rhythm, time interval from collapse to ROSC (if witnessed), as well as patient comorbidities and home medications.

In-hospital data reported included admission time (day defined as 8 am to 4 pm based on staffing and probable cause of cardiac arrest [cardiac vs. non-cardiac]). Our department is staffed by 3 attending and 7 resident physicians, as well as 8 nurses (patient:nurse ratio 2:1) from 8 am to 4 pm, and 1 attending and 3 residents (4 on weekends and holidays) from 4 pm to 8 am. Recognizing potential limitations of arbitrary definitions of day and night, we conducted a sensitivity analysis to assess the robustness of our findings by using an alternative definition of day admission, defined as admission between 8 am and 7:59 pm on the basis of prior publications,^10^ as well as using a more stringent definition of nighttime from 11 pm to 7 am.

We provided postresuscitative care according to established guidelines and local standardized protocols.^5,21^ To assess the quality of post arrest care, we calculated the proportion of patients who received IH at day vs. night, as well as time from hospital admission to initiation of hypothermia. We also calculated the proportion of patients undergoing revascularization (PCI), as well as the time from admission to PCI. Physiologic variables, including body temperature, pH, lactate level, glucose level, and Troponin T, were recorded at admission, 6 and 12 hours after ROSC, respectively.

Survival and neurologic outcome were assessed 12 months after ROSC using the Cerebral Performance Category (CPC) scale.^22^ Good neurologic outcome was defined as CPC 1 (good neurologic function) or 2 (moderate disability) on the 5-point CPC scale. Patients in CPC categories 3 to 5 (severe disability, vegetative state, or death) and patients who never regained consciousness because of necessary analgosedation during the study period or before death were classified as poor neurologic outcome.

### Statistical Analysis

Continuous data are presented as means and standard deviations or medians and interquartile ranges (interquartile range [IQR], 25%–75%) as appropriate. Categorical data are presented as counts and relative frequencies. Student *t* test, Mann–Whitney *U* test, or χ^2^ tests were used for comparisons between day and night admissions. We constructed a logistic regression model to assess the odds of survival at 12 months, adjusting for age, sex, initial rhythm, comorbidity, arrest location, cause of arrest, and daytime admission. A *P* value of <0.05 was considered statistically significant. Statistical analyses were performed using STATA software (Statacorp, College Station, Texas, USA).

## RESULTS

### Patient Characteristics

**FIGURE 1:**
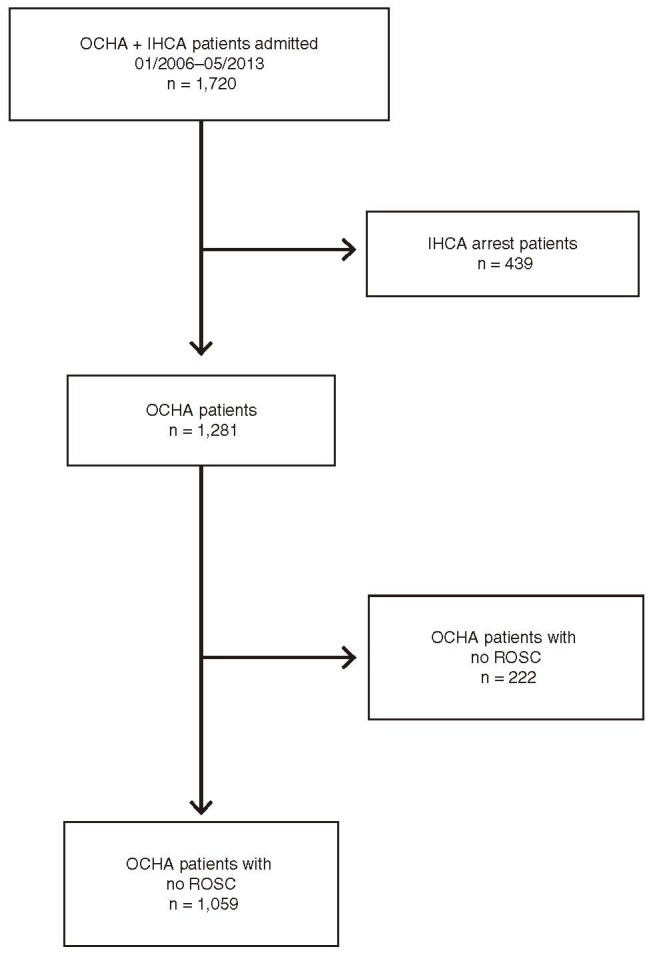
Overview of analysis cohort. IHCA = in-hospital cardiac arrest, OHCA = out-of-hospital cardiac arrest, ROSC = return of spontaneous circulation.

**FIGURE 2:**
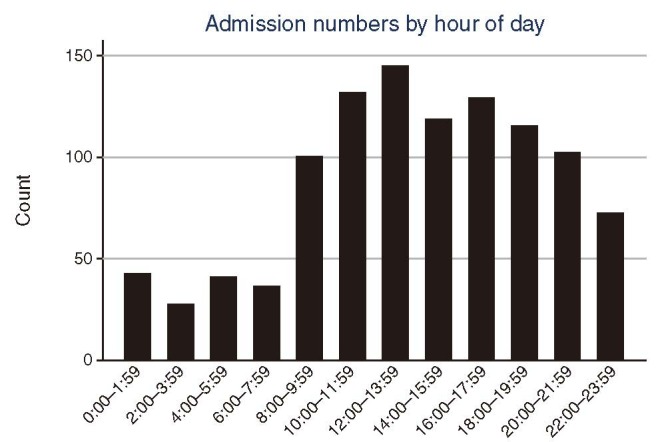
Volume of admissions by hour of day.

Table 1 compares characteristics of patients hospitalized after OHCA with ROSC. Of 1720 patients admitted during the study period, we identified 1059 patients equal to or older than 18 years of age who regained ROSC after an OHCA (Figure 1). Of those patients, 490 (46%) were admitted between 8 am and 4 pm (day) and 569 (54%) were admitted during night (4:01 pm to 7:59 am) (Figure 2). Patients admitted at night were slightly younger compared with those admitted during daytime (56 vs. 60 years, *P* < 0.001). The proportion of males was similar in both groups (74% vs. 74%, *P* = 0.96). Mean body mass index (BMI) was similar in both groups (27 for daytime vs. 27 for nighttime admissions, *P* > 0.05), as was the proportion of current smokers (30% for daytime vs. 30% for nighttime admissions, *P* > 0.05). The overall burden and distribution of comorbid conditions were similar among patients admitted during daytime vs. nighttime (53% of daytime admissions vs. 54% of nighttime admissions had at least 1 documented comorbid condition, *P* = 0.62). Most prevalent were hypertension, coronary artery disease, diabetes, and previous history of myocardial infarction (Table 1). Overall, 28% of patients had documented home medications, and the proportion of patients who were noted to take antiplatelet drugs or antilipid drugs did not differ by day vs. night (8% vs. 7% for antiplatelet therapy and 6% vs. 5% for antilipid therapy, respectively; both *P* > 0.05).

**TABLE 1:**
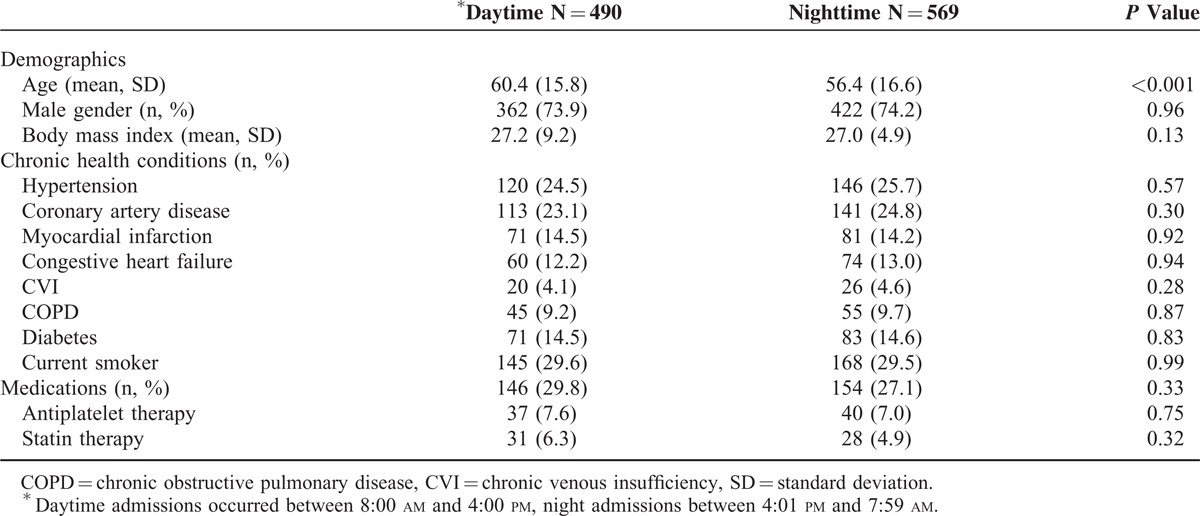
Patient Baseline Characteristics

### Prehospitalization Arrest Characteristics

The vast majority (83%) of arrests were witnessed, with no difference between day and night admissions (Table 2). Patients admitted at night were more likely to have had arrested at home (53% vs. 37%, *P* < 0.001), whereas patients admitted during the day were more likely to have had arrested in public places (49% vs. 38%, *P* < 0.01). Approximately 10% of patients arrested during transport to hospital (11% during day vs. 8% at night). Cardiac and pulmonary etiologies accounted for nearly 80% of all arrests, and there was a trend toward more cardiac etiology in patients admitted during the day (69% day vs. 62% night, *P* = 0.09), and more pulmonary etiology in patients admitted during nighttime (15% night vs. 11% day; *P* = 0.09). This was reflected by a higher proportion of ST elevation myocardial infarctions (STEMI) among daytime admissions (31% day vs. 26% night, *P* = 0.05). Bystander CPR was performed in approximately a third of cases, with similar proportions between day and nighttime admissions (39% day vs. 37% night, *P* = 0.49). Overall, the majority of patients (57%) initially had a shockable rhythm (ie, ventricular fibrillation or pulseless ventricular tachycardia). We observed a trend toward more shockable initial rhythms in patients admitted during daytime (61% day vs. 54% night) and more nonshockable rhythms (asystole and pulseless electrical activity) during nighttime admissions (41% night vs. 33% day, *P* = 0.07). The number of shocks applied, cumulative dose of epinephrine administered before ROSC, and proportion of patients on vasopressor therapy immediately after admission did not differ between daytime and nighttime admissions (Table 2). Among those who had a witnessed cardiac arrest, mean time from arrest to ROSC was similar in patients admitted during day and night (20 ± 14 min at day vs. 20 ± 14 min at night, *P* = 0.58). Similarly, the time from ROSC to hospital admission did not differ between day and night admissions (29 ± 19 min vs. 31 ± 19 min, *P* = 0.31). In addition, physiologic variables at admission, including core body temperature, pH, lactate level, blood glucose level, and potassium level, did not differ between day and night admissions (Table 3).

**TABLE 2:**
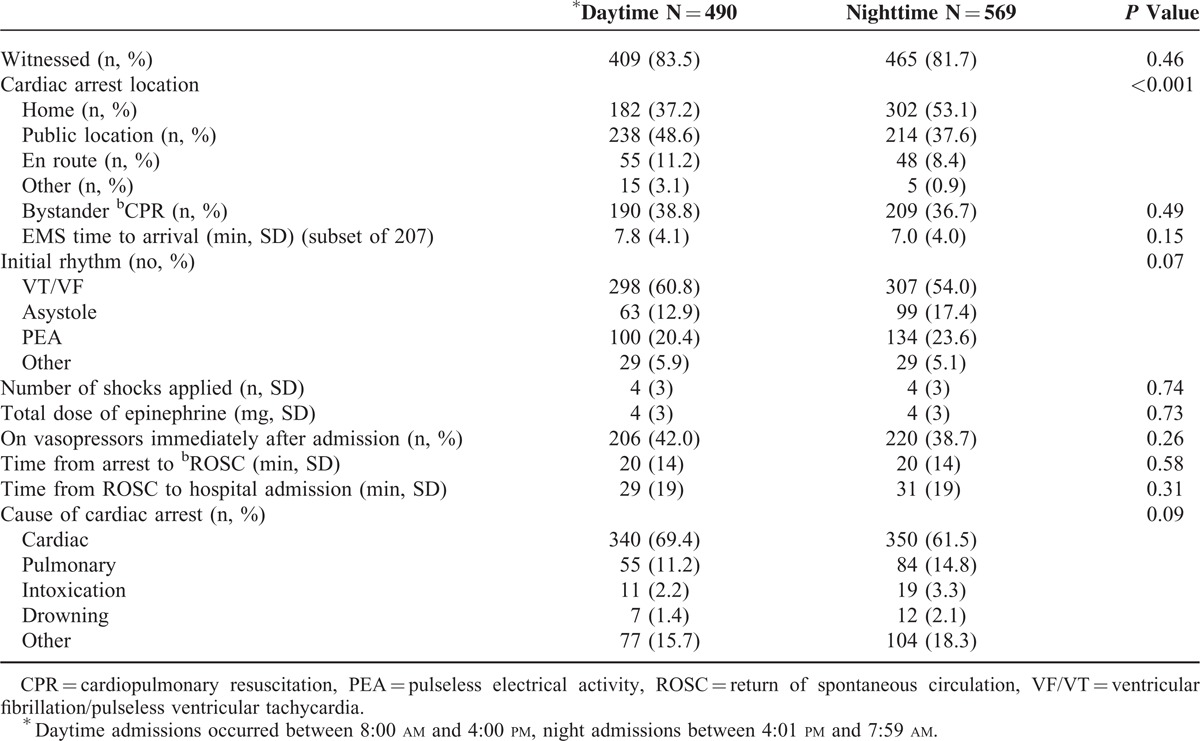
Prehospitalization Arrest Characteristics

**TABLE 3:**
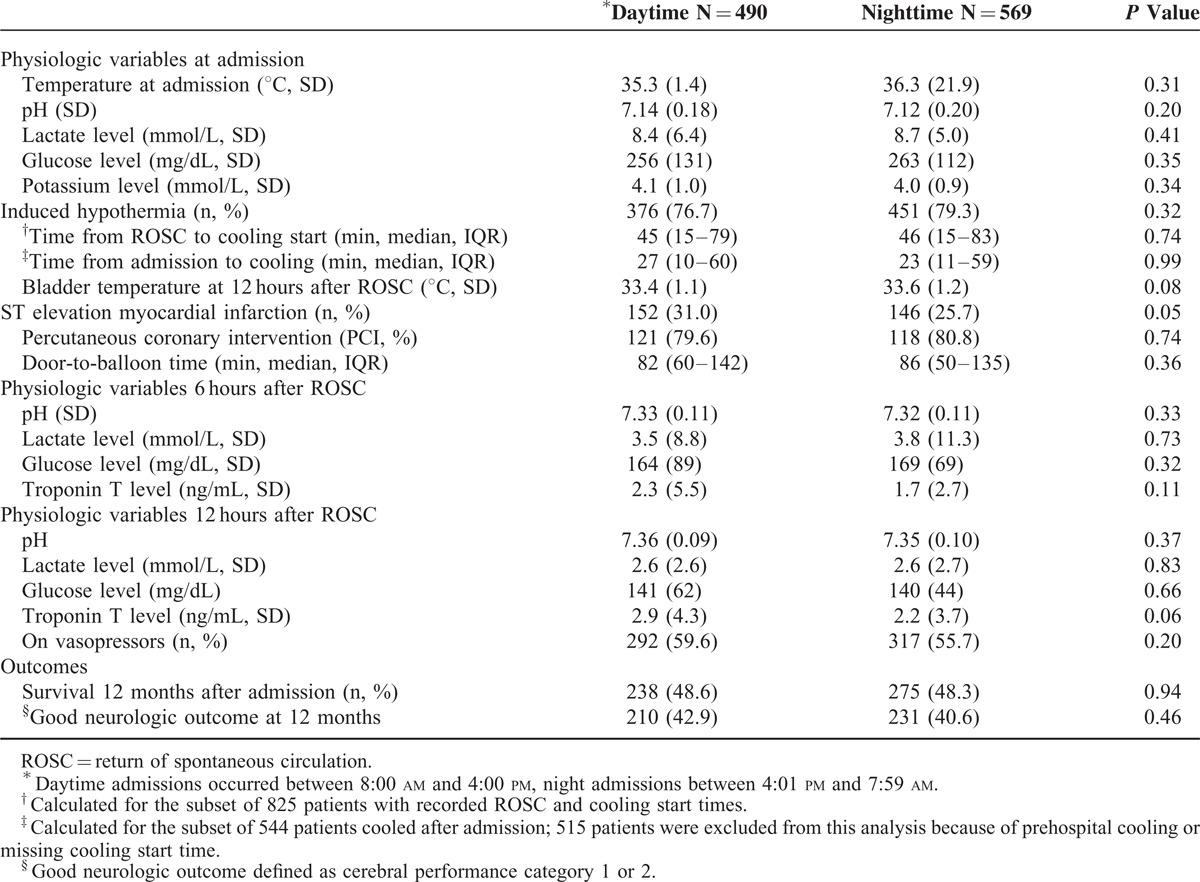
Posthospitalization Arrest Characteristics

### Posthospitalization Arrest Characteristics

The vast majority of patients (827, 78%) were treated with IH (77% day vs. 79% night, *P* = 0.32). For the subset of 825 patients with documented ROSC and cooling start times, median time from ROSC to initiation of hypothermia was 45, confidence interval (CI): 15–79 minutes during daytime and 46, CI: 15–83 minutes at night (*P* = 0.51). Similarly, for the subset of 544 patients whose cooling was initiated after admission to our facility (ie, did not receive any prehospital cooling) and had a recorded cooling start time, median time from admission to cooling did not differ between day and night admissions (27, CI: 10–60 minutes during day vs. 23, CI: 11–59 minutes at night, *P* = 0.99). Similar bladder temperatures in both groups 12 hours after admission suggest equally effective hypothermia in both groups (33.4 ± 1.1°C for patients admitted during daytime vs. 33.6 ± 1.2°C for patients admitted during night).

Among the 298 patients with STEMI, 239 (80%) received PCI (80% of day admissions vs. 81% of night admissions, *P* = 0.74). Median door-to-balloon time was similar in both groups (82 minutes, IQR: 60–142 during daytime and 86 minutes, IQR: 50–135 during nighttime, respectively, *P* = 0.36). Physiologic variables 6 and 12 hours after admission, including body temperature, pH, lactate level, blood glucose level, and myocardial enzymes, did not differ significantly between daytime and nighttime admissions (Table 3).

These results remained unchanged using an alternative definition of daytime from 8 am to 7:59 pm based on prior publications,^10^ as well as using a more stringent definition of nighttime from 11 pm to 7 am.

### Outcomes

**FIGURE 3:**
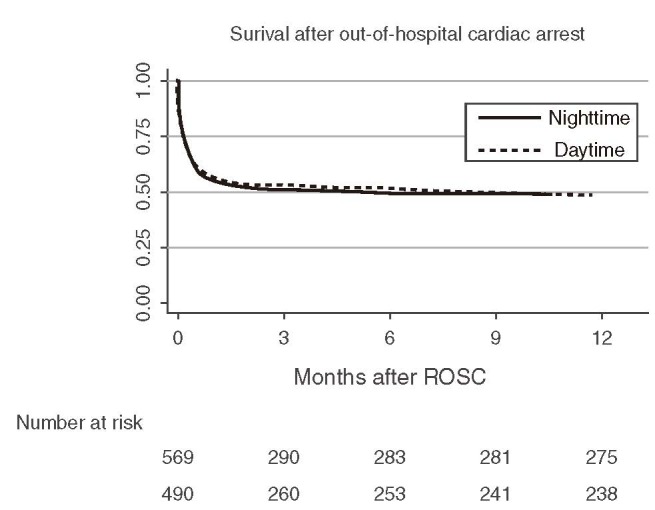
Kaplan–Meier curve for comparison of 12-month survival between daytime and nighttime admissions after out-of-hospital cardiac arrest. ROSC = return of spontaneous circulation.

Survival after 12 months was similar in patients admitted during day and nighttime (49% and 48%, respectively, *P* = 0.94; Table 3 and Figure 3). The probability of survival at 12 months remained unchanged after adjusting for age, sex, initial rhythm, arrest location, and comorbid conditions (OR, 1.01; 95% CI, 0.76–1.31, *P* = 0.96 for daytime vs. nighttime admissions). Of all patients admitted, 441 (42%) had a favorable neurologic outcome at 12 months (43% for daytime admissions vs. 41% for nighttime admissions, *P* = 0.46). No patients were lost to follow up.

## DISCUSSION

In contrast to previous studies,^10–12^ we did not observe any differences in survival or functional outcomes between patients admitted at daytime and nighttime (defined as 4 pm to 7:59 am), respectively. In addition to accounting for prehospital components of care and patient characteristics, we investigated quality of in-hospital post arrest care between patients admitted at daytime and nighttime.

Overall, baseline patient and prehospitalization characteristics were similar between daytime and nighttime admissions with the exception that patients admitted at night were slightly younger than patients admitted during the day, and nighttime arrests more commonly occurred at home. These differences are consistent with prior studies.^10–12,23^ The first blood gas after admission, analyzed within 15 minutes after admission, reflected the overall equality of prehospital care between daytime and nighttime admissions (Table 3).

Several studies reported poorer quality of time-sensitive treatments during off hours as compared with routine hours. For instance, Campbell et al^24^ reported delays in receiving a CT scan, admission to a dedicated stroke unit, and reduced odds for thrombolytic therapy in a British stroke cohort presenting off hours vs. regular hours. Similarly, a Dutch study reported nearly 2-fold higher angioplasty failure and mortality rates in patients with acute myocardial infarction treated during off-hours.^25^ Current guidelines for post arrest care recommend mild IH for 12 to 24 hours after ROSC.^26^ Both groups received cooling within guideline definitions and without difference between groups. In our cohort, median time from admission to cooling start was 25 minutes and did not differ between day and night.

Consistently, mean core body temperature 12 hours after ROSC did not differ between both groups, implying the same quality care during all times of the day. Other indicators of quality of care, such as glucose levels and lactate levels (as indicator of adequate resuscitation), did not differ between both groups 12 hours after ROSC. In addition, median door-to-balloon time in the subset of patients treated with PCI was close to 90 minutes and did not differ between day and night admissions. These time intervals comply with the definition of “early PCI” after cardiac arrest^17^ and current guidelines for timely PCI in STEMI patients (door-to-balloon time less than 90–120 minutes).^26^

Our results differ from previous studies that reported worse outcomes for OHCA patients admitted at night.^10–12^ These differences may be explained by variations in the quality of post arrest care. For example, our institution meets the American Heart Association criteria of a cardiac center of excellence^27^ and includes an integrated intensive care unit, in which patients are treated up to 24 hours after admission. It is staffed with highly trained critical care nurses and emergency physicians with additional training in critical care medicine. Previous studies have shown a positive volume–outcome relationship for centers treating at least 40 cardiac arrest patients per year.^14,28,29^ In perspective, our center treats approximately 250 cardiac arrests annually. Furthermore, 24-hour PCI availability is associated with improved survival after cardiac arrest.^30^ Hence, our data support concentration of post arrest care in highly specialized cardiac arrest centers that have the infrastructure to treat cardiac arrest patients proficiently around the clock.

### Limitations

There are several limitations in this study. First, it is an observational retrospective single-center study. Although baseline characteristics and prehospital characteristics were similar to those of prior multicenter studies,^10–12^ our outcomes may not be applicable to other institutions with less proficient post arrest care. Nonetheless, our department treats around two-thirds of all hospitalized cardiac arrest patients in the 2 million metropolitan area of Vienna.^19^ Second, we excluded patients whose resuscitation efforts were futile and who deceased before hospital arrival. However, we expect that any introduced bias would equally affect day and night time admissions.

## CONCLUSION

Patients admitted to our specialized resuscitation center after OHCA were equally likely to receive timely high-quality postresuscitation care irrespective of time of day. Similarly, survival and good neurologic outcomes at 12 months did not differ between day and night admissions. Previously reported circadian differences in OHCA outcomes may be because of variation in the quality of postresuscitation care provided at nonspecialized centers. Our results may support the concept of specialized post arrest care centers.
